# Comparison of feasibility, time consumption and costs of three virtual planning systems for surgical correction of midfacial deficiency

**DOI:** 10.1186/s40902-020-00284-1

**Published:** 2021-01-07

**Authors:** Katrin Willinger, Godoberto Guevara-Rojas, Julia Cede, Kurt Schicho, Tanja Stamm, Clemens Klug

**Affiliations:** 1 University Clinic of Cranio- and Maxillofacial Surgery, Medical University of Vienna, Vienna, Austria; 2 University of Applied Sciences FH Campus Wien, Vienna, Austria; 3 Center for Medical Statistics, Informatics and Intelligent Systems (CeMSIIS), Medical University of Vienna, Vienna, Austria

**Keywords:** Orthognathic surgery, Intraoral quadrangular Le Fort II osteotomy, Virtual surgical planning, CAD/CAM technology, Time and cost in corrective maxillofacial surgery

## Abstract

**Background:**

Today virtual surgical planning (VSP) is a standard method in maxillofacial corrective surgery and is the key to reach satisfactory esthetic outcomes. The purpose of this study was to evaluate usability of three established virtual surgical planning software applications by comparing feasibility, time consumption, and costs in a standardized workflow for a modified intraoral quadrangular Le Fort II osteotomy (IQLFIIO).

**Results:**

A cross-sectional study was performed based on retrospective and re-planned data of patients with midfacial deficiency treated by modified IQLFIIO, using three software applications: IPS Case Designer ®, Dolphin Imaging ®, and ProPlan CMF ®. Feasibility: All evaluated steps of the VSP procedure could be successfully performed in all three evaluated applications. In all software packages, it was possible to design the surgical splints with CAD/CAM technology. Working time: The mean value of time needed was IPS Case Designer ®, 36.5 min; Dolphin Imaging ®, 33.6 min; ProPlan CMF ®, 45.5 min. We found statistical significant difference between ProPlan CMF ® and Dolphin Imaging ® (*p* value, 0.02). Costs: Asset costs for acquiring the software, license fee, license possibilities, paying for support services, and service contracts were evaluated and are found in similar ranges.

**Conclusion:**

All three tested software applications are usable for virtual planning of an IQLFIIO and splint production by CAD/CAM technology. Successful movement of bone segments and overlaying soft tissues proved feasibility. Time consumption and costs were found in similar ranges.

## Introduction

Today virtual surgical planning (VSP) is a standard method in facial corrective surgery and is the key to reach satisfactory esthetic outcomes. In the last years, VSP and CAD/CAM (computer-aided design/computer-aided manufacturing) technology have revolutionized the planning process and can also be used as a patient communication tool in corrective maxillofacial surgery. VSP includes the simulation of surgical movements, bones as well as their overlaying soft tissue, and is very helpful to imagine post-intervention positions especially of midface changes.

Whereas a Le Fort I osteotomy is today’s gold standard for the correction of the occlusion by skeletal movement of the lower maxilla, intraoral quadrangular Le Fort II osteotomy (IQLFIIO) enables the advancement of the entire midface. The medical indication for an IQLFIIO is an angle class III malocclusion paired with midfacial deficiency including the infraorbital region. Affected patients often suffer from social discrimination because of a stigmatizing effect of midfacial deficiency. A concave facial profile is highly associated with unfavorable characteristics [1]. IQLFIIO was first described by Keller and Sather in 1987 [2] as a suitable method for the correction of the midface region. Recently, a technically modified IQLFIIO was reported to achieve reliable midfacial advancement with a reduced morbidity compared to the original method [3].

In literature, we found several studies comparing conventional planning (cephalogram and dental casts) with virtual 3D planning. Mostly, they focus on accuracy and applicability. A few studies also compared time and costs of conventional 2D and 3D planning [4–7].

Today surgical splint fabrication using CAD/CAM technology is an established method [4, 8]. For this purpose, various virtual surgical planning applications were generated over the last years. Examples for widely used software applications are IPS Case Designer ®, Dolphin Imaging ®, and ProPlan CMF ®.

## Material and methods

### Aim

The purpose of this study was to evaluate the usability of three established VSP software applications (IPS Case Designer ®, Dolphin Imaging ®, and ProPlan CMF ®) regarding the virtual planning steps of midfacial correction using modified IQLFIIO. Specific aims were to compare feasibility, time consumption, and costs in a standardized workflow.

### Design

Based on retrospective data, we performed a cross-sectional study and re-planned all patients with midfacial deficiency treated by modified IQLFIIO and BSSO (bilateral sagittal split osteotomy) at our institution between April 2013 and December 2018 using the three software applications: IPS Case Designer ®, Dolphin Imaging ®, and ProPlan CMF ®.

The study sample was recruited from a consecutive series of patients with midfacial deficiency treated by modified IQLFIIO and BSSO. Inclusion criteria were midfacial deficiency and skeletal class III malocclusion as well as a fulfilled protocol with pre-surgical and post-surgical orthodontic treatment. The surgical treatment had to be completed at the time point of the beginning of the study. Exclusion criteria were missing or poor quality of available pre-surgical computer tomography (CT) data.

This study was approved by the ethics committee of the authors’ institution (No. EK 1775/2017) and performed according to the Declaration of Helsinki and the guidelines for Good Clinical Practice (GCP).

### Data acquisition—virtual surgical planning

In this retrospective study, all cases were re-planned by VSP with three different software application systems. The pre-intervention data were obtained from existing CT or cone-beam CT (CBCT) examinations of the head. VSP was done with the software applications IPS Case Designer ® (KLS Martin Group), Dolphin Imaging ® 11.95 (Patterson Dental Supply, St. Paul, MN), and ProPlan CMF ® (Materialise). A routine workflow for VSP for IQLFIIO was performed. This included data import of Digital Imaging and Communications in Medicine (DICOM) datasets, rendering a 3D image of the head, setting the intervention cut lines for IQLFIIO, calculating the surgical intervention of the 3D model for hard and soft tissue. The last step was virtual model surgery by moving the maxilla including the infraorbital region into the target position.

All VSP were performed with a personal computer—Dell Inspiron 15 5000 Notebook (Intel Core i5-7500 U, 16 GB RAM, 256 GB SSD, AMD Radeon R7 M445) by one experienced person who had been trained on all three applications.

### Variables

To evaluate and compare feasibility, the following steps in the process of VSP of an IQLFIIO were evaluated: (i) preselect a IQLFIIO tool, (ii) setting of cut lines on defined IQLFIIO landmark positions (maxilla, infraorbital rim, anterior nasal spine), (iii) 3D rendering of the segment to be moved, (iv) act of moving to the target position, and (v) possibility to design a splint.

In order to establish a standardized and realistic procedure, post-surgical intervention results were defined as the target position for the VSP. Distances between pre- and post-operative landmarks were measured in a previous step to import exactly these movements into the VSP system. These data were obtained by image fusion using Materialise Mimics ® Research 21.0 (Mimics-Materialise NV Belgium). These measurements were used to set the target position in all three compared VSP systems.

The IQLFIIO cut lines were set in positions corresponding to the post-operative CT scans. The 3D rendered model was cut exactly along these lines. The selected IQLFIIO part was moved into the target position by manual input of the known distances. All the measurements and VSP have been done by one researcher who has been experienced and trained in using these software tools consequently.

Regarding time used for the VSP, the time of the surgeon working with the PC was measured for all steps of the VSP procedure in minutes (min).

Regarding costs, asset costs for acquiring the software application, license fee, license possibilities, paying for support services, service contracts, and costs for splint production in cooperation with the VSP system were covered by contacting the company. Costs were calculated in dollars ($) without value-added tax.

### Data analysis

For characterizing the study, cohort descriptive statistics was used. All data were recorded in Microsoft Excel 2017.

The purpose of this study was to evaluate the usability of three established VSP software applications (IPS Case Designer ®, Dolphin Imaging ®, and ProPlan CMF ®) regarding the virtual planning steps of midfacial correction using modified IQLFIIO. Specific aims were to compare feasibility, time consumption, and costs in a standardized workflow.

For the graphical analysis of time consumption, a boxplot graphic was created. For comparison time consumption, a post hoc analysis with a Tukey range test was performed.

Statistical analysis was performed using the open source software R Project R 3.1.1. The costs are outlined in tables.

## Results

VSP with three systems was performed with the radiological data (CT and DVT) of 19 skeletally mature patients aged between 18 and 37 years (5 female, 14 male, mean age 22 years). All patients were part of a consecutive series treated by IOQLFIIO and BSSO for class III malocclusion and midfacial deficiency. All patients were Caucasian and met the inclusion criteria.

### Feasibility

All evaluated steps of the VSP procedure, including (i) preselect a IQLFIIO tool, (ii) setting and definition IQLFIIO cut lines, (iii) 3D rendering of the segment to be moved, (iv) moving the IQLFIIO part to target position could be successfully tested in all three evaluated applications in all 19 cases.

**Fig. 1:**
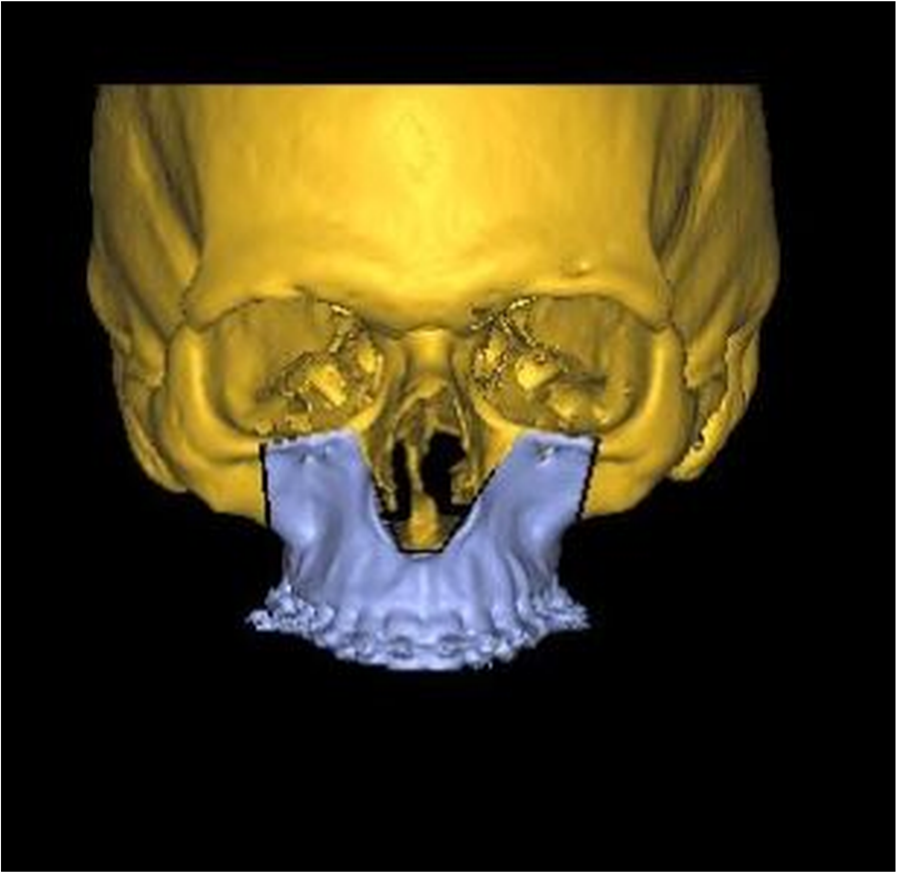
IQLFIIO segment planned in ProPlan CMF ®

**Fig. 2:**
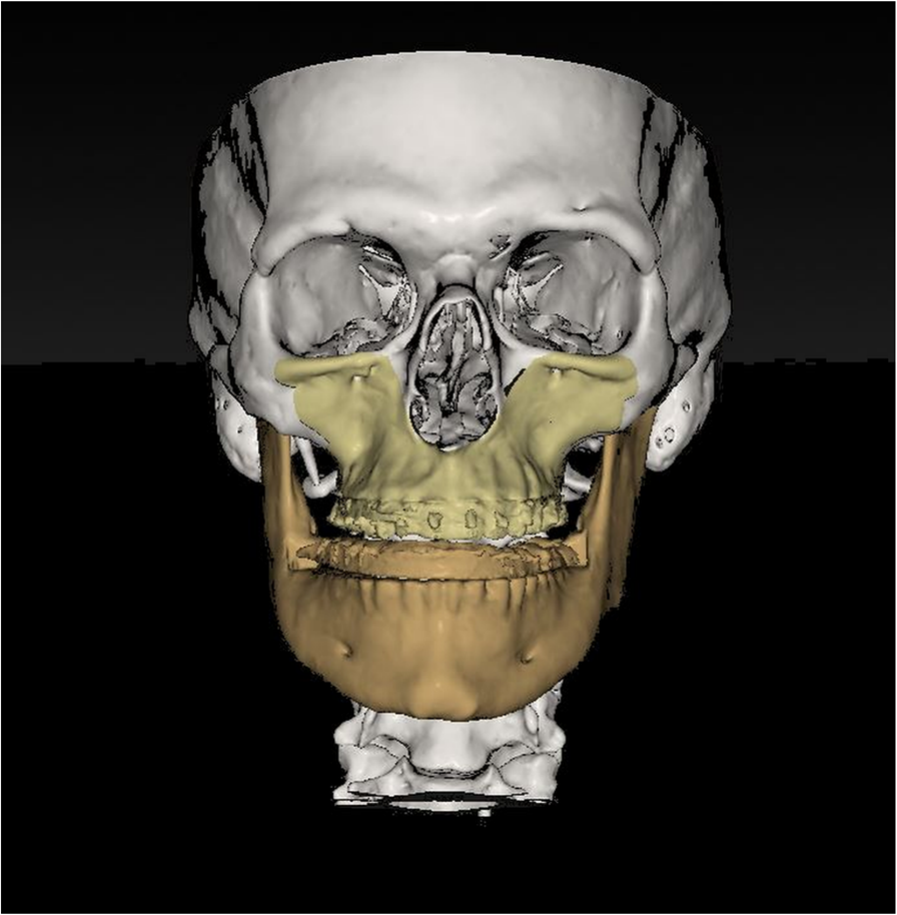
IQLFIIO segment and the Mandible in the target occlusion in IPS Case Designer ®

**Fig. 3:**
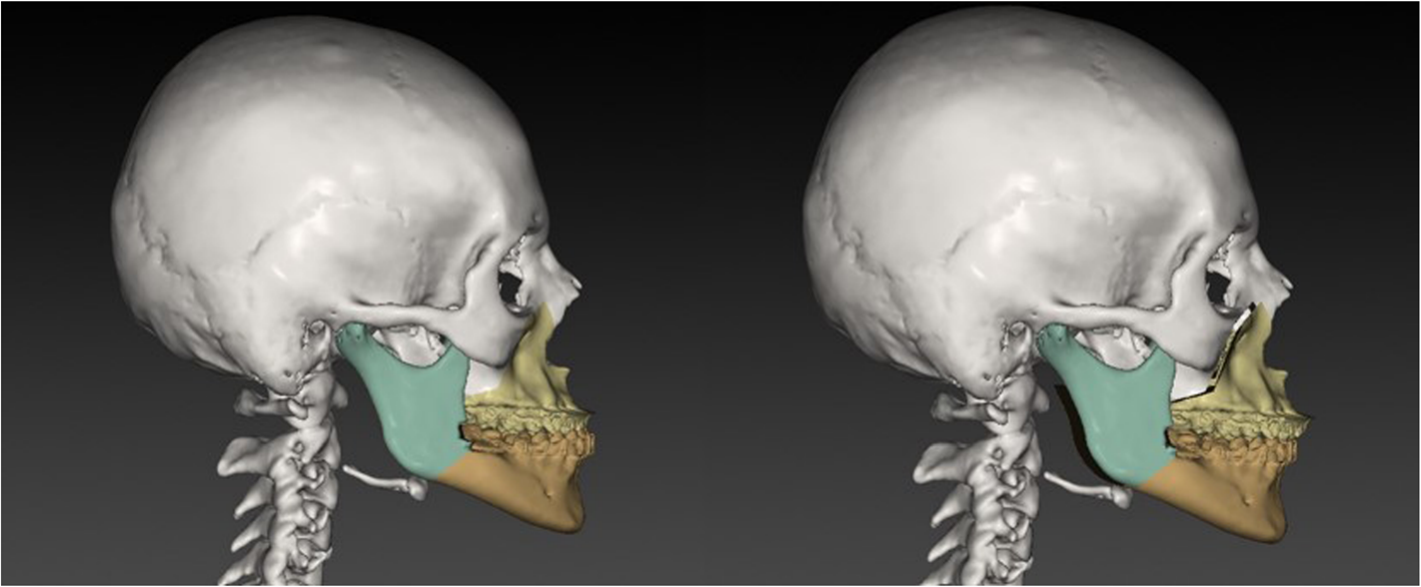
IQLFIIO segment and the Mandible in a lateral view in the initial and target occlusion in IPS Case Designer ®

**Fig. 4:**
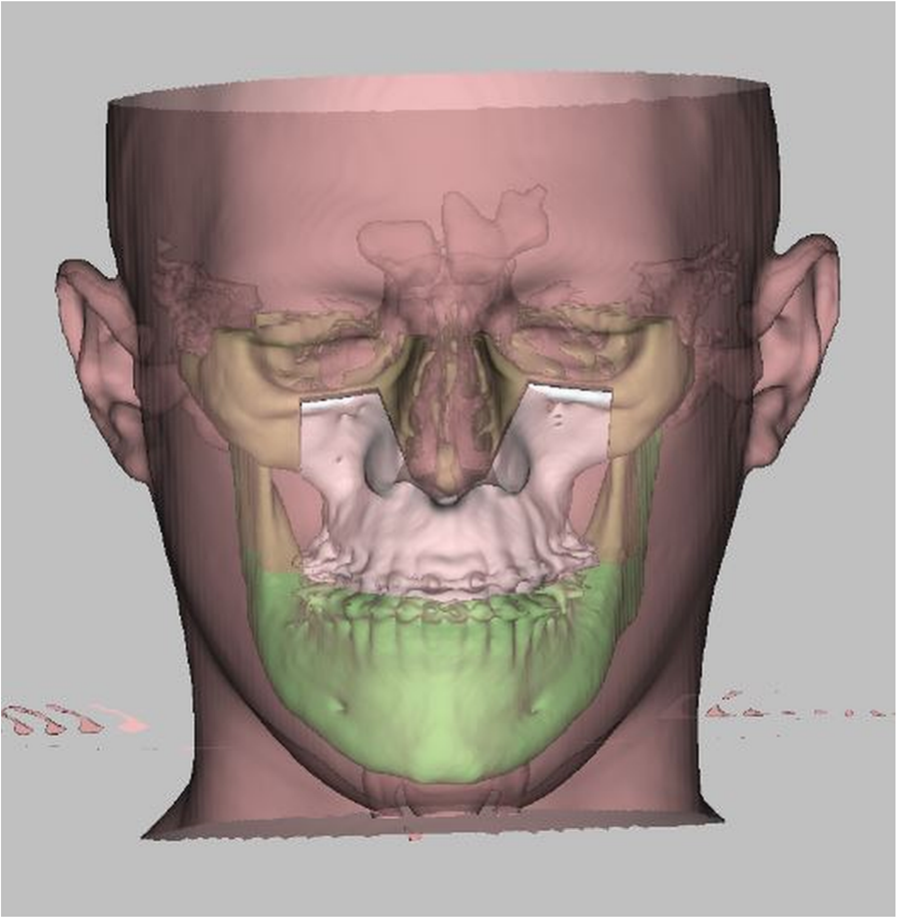
Simulation of the treatment result with transparent overlaying soft tissue in Dolphin Imaging ®

**Fig. 5:**
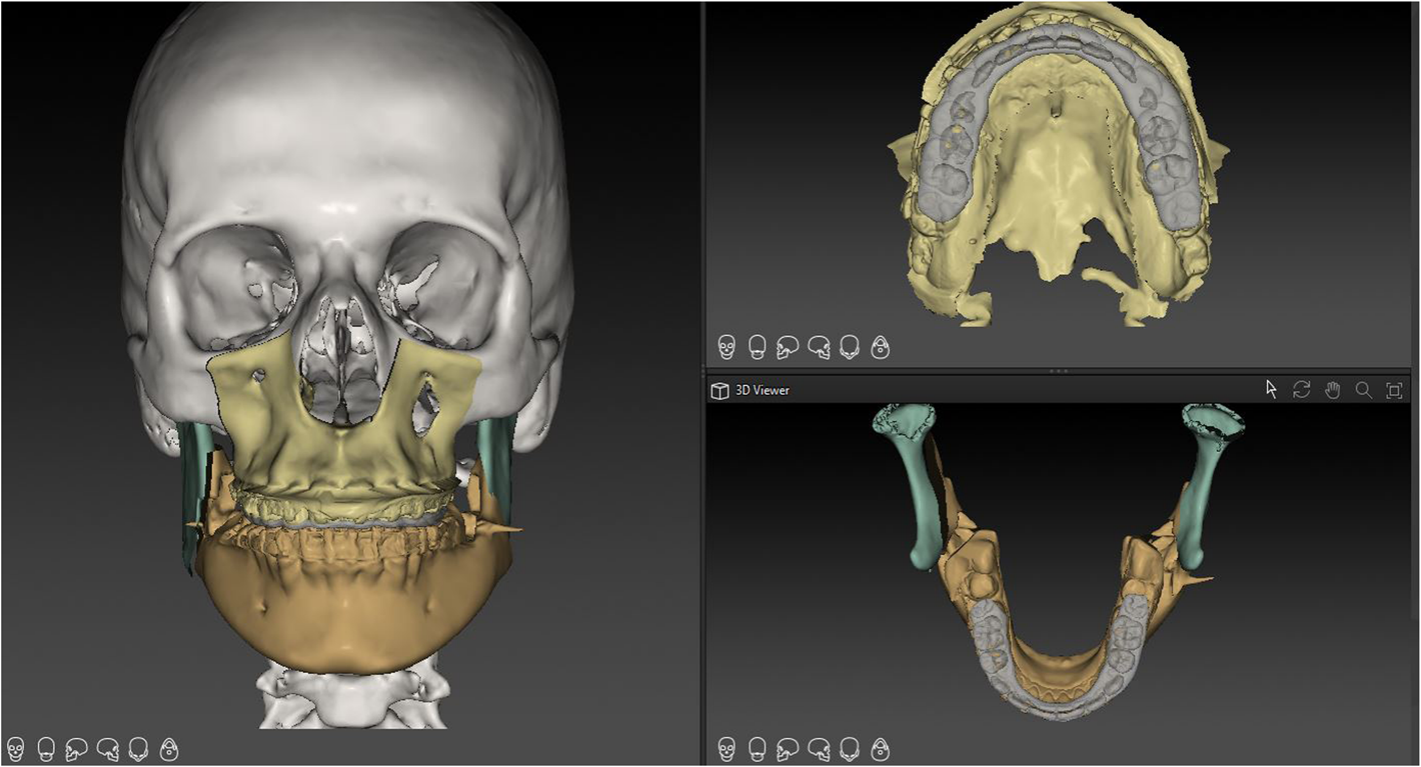
Surgical splint design with CAD/CAM technology using the software applications IPS Case Designer ®

Differences between the three tested software applications among another were found: (i) Only the system ProPlan CMF ® offers a tool to select Le Fort II osteotomy. A VSP of the IQLFIIO performed with this system is depicted in Fig. 1. In IPS Case Designer ® and Dolphin Imaging ®, only the Le Fort I intervention type for the upper jaw surgery was selectable. (ii) It was possible to manually set the cut lines in the Le Fort II level in the IPS Case Designer ® and Dolphin Imaging ®. When using ProPlan CMF ®, a tool called “Mimics” has to be used prior to planning for rendering and maxillary and mandibular or the whole Midface region segmentation. It is required for setting the surgical cut lines in a second step. (iii) 3D rendering was possible in all three systems. A VSP of the IPS Case Designer ® system is seen in Figs. 2 and 3. (iv) To finish the VSP process, moving the segmented part to the target position was also possible. As well as to visualize soft tissue changes—seen in Fig. 4 (Dolphin Imaging ®). (v) Surgical splint design using with CAD/CAM technology was also possible in all three software applications. An example is given in Fig. 5.

### Working time

For the VSP system IPS Case Designer ®, the mean value of time needed was 36.5 min (median, 32 min; minimum, 20 min; maximum, 60 min).

For the VSP system Dolphin Imaging ®, the mean value of time needed was 33.6 min (median, 30 min; minimum, 20 min; maximum, 60 min).

For the VSP system ProPlan CMF ®, the mean value of time needed was 45.5 min (median, 43 min; minimum, 25 min; maximum: 63 min).

**Fig. 6:**
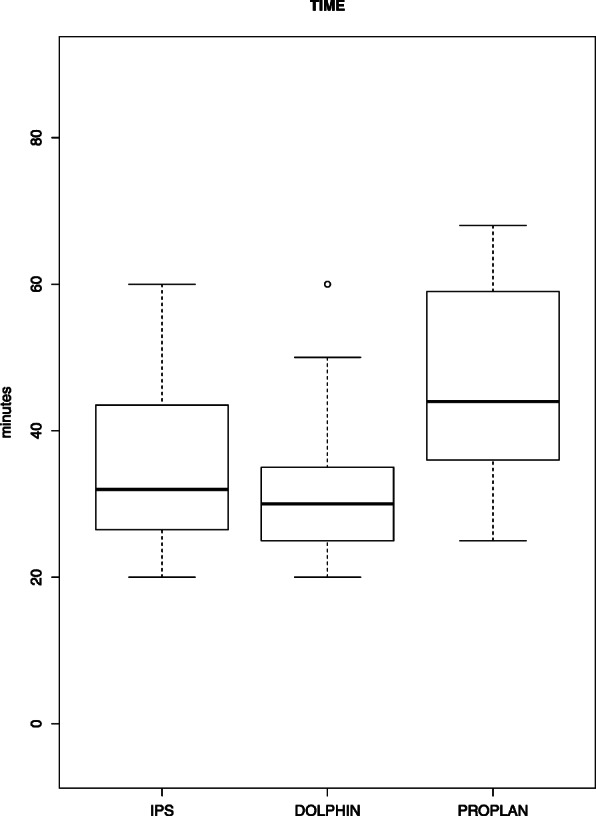
Boxplot of time consumption in minutes for the software applications IPS Case Designer ®, Dolphin Imaging ® 11.95 and ProPlan CMF ®

Also seen in Fig. 6.

To compare the three systems regarding working time, we performed a post hoc analysis. Therefore, a Tukey range test was used. We found a statistical significant difference between ProPlan CMF ® and Dolphin Imaging ® (*p* value, 0.02). There was no statistical significance found between the IPS Case Designer ® and Dolphin Imaging ® (*p* value, 0.7) and ProPlan CMF ® and IPS Case Designer ® (*p* value, 0.09). The difference measured in minutes between the means of IPS Case Designer ® and Dolphin Imaging ® was 2.9, between ProPlan CMF ® and Dolphin Imaging ® 11.9, and between ProPlan CMF ® and IPS Case Designer ® 9.

### Costs

The three tested VSP systems are available in different software packages. The costs are in a similar range. Dependent on which package to choose, asset costs for acquiring the software, license fee, license possibilities, paying for support services, and service contract costs differ. The authors decided to show these results in tables. For the VSP system IPS Case Designer ®, see Table 1.

**Table 1:** Overview of the costs for the VSP system IPS Case Designer ®

	1	2	3	4
IPS Case Designer ®	Office	Hospital	Academic	Test
Asset costs for acquiring	10,900 $	14,900 $		Free
License	2 users, unlimited	6 users, unlimited	6 users, unlimited	1 user, 90 days
Service/update/support	1 year/afterwards: 3,000 $ p.a.	1 year/afterwards: 3,000 $ p.a.	1 year/ afterwards: 3,000 $ p.a.	Only support

Costs of the VSP system Dolphin Imaging ® are shown in Table 2.

**Table 2:** Overview of the costs for the VSP system Dolphin Imaging ®

	1	2	3
Dolphin Imaging ®	Business	Business +	Academic
Asset costs for acquiring	25,800 $	= extension of 1	25,800 $
License	1 user, perpetual	1,900 $ for server, 3,450 $ for additional user	3 users, annually
Service/update/support	1 year/afterwards: 3,500 $ p.a.	1 year/afterwards: 3,500 $ p.a.	1 year/afterwards: 3,500 $ p.a.

In the Dolphin Business version, the license is perpetual. The annual fee is not mandatory, but updates or support run out if not paid.

In the VSP system ProPlan CMF ®—see Table 3—there is no initial software training included in the price. But it is possible to buy a training session.

**Table 3:** Overview of the costs of the VSP system ProPlan CMF ®

	1	2	3
ProPlan CMF ®	Dysgnathia local	Dysgnathia floating	Test
Asset costs for acquiring	8,412 $ p.a.	12,617 $ p.a.	Free
License	1 user, 1 year	6 users, 1 year	1 user, 14 days
Service/update/support	1 year	1 year	Only support

Costs for splint production in cooperation with the current company were also assessed. The files could be exported for free and the splints can be built in a 3D printer. The VSP system IPS Case Designer ® from the KLS Martin Group company was the only application software which offers the possibility to send the splint files during the planning process via data transfer for production. The splints will be sent back per post. Costs for splint fabrication were 182.25 $ per splint.

## Discussion

This paper aims to assess the usability of three different software applications for VSP in orthognathic surgery. The authors defined usability as feasibility, time consumption for digital working process, and costs for acquiring a software package. IQLFIIO was chosen because the investigators assume that if it was possible to plan a more complex surgical intervention, it would also be possible to plan any other osteotomies. For orthognathic surgeons, software applications should not only allow to work with cutlines for standardized osteotomies (i.e., Le Fort I osteotomy only); they should also enable the surgeon to set individualized cutlines. Assessment of feasibility also included the process of rendering, segmentation, and segment movement. It was important to evaluate the concomitant change of the overlaying midfacial soft tissue.

For all three tested applications, these steps were performed successfully in all cases, but differences were found. ProPlan CMF ® is the only one, which offers a Le Fort II tool. In the other two applications, a manual modification of the Le Fort I cut lines has to be performed. Using ProPlan CMF ® requires prior rendering and segmentation with the application Mimics ®, a tool provided by the same company. The number of crash events of the PC operating system was also reported for evaluation of usability (IPS Case Designer ®—1; Dolphin Imaging ®—4; ProPlan CMF ®—3). There were exclusively non-reproducible software bugs, which required a reboot of the system. The VSP steps were all stored in a temporary cache, so all software systems are suitable to be used in a routine work. The authors are well aware that continuous improvement by software updates from the companies make this point rather a snapshot than a reliable result. Never the less, it illustrated that digital planning still is technically demanding and PC crashes do occur.

As described in literature, the investigators also found out, that successful model surgery and the use of CAD/CAM technology for splint production require a good quality of pre-surgical 3D data of the patient [8–10]. A satisfying surgical outcome depends on the pre-surgical VSP and the exact transfer of planning steps to surgical intervention [5]. Several studies about validation and accuracy of 3D printed splints are found in literature [4, 11, 12]. This is similar for all software applications but not the topic of this work.

Generally, splint design is possible in all three evaluated software applications. They offer the possibility to export surface files (STL format) for 3D printing. This is a free option. Only when working with the application IPS Case Designer ®, it is possible to order splints from the company directly. However, processing time and time of delivery of the produced splints have to be considered in the individual workflow. The two other software applications require a 3D printer in the office. Alternatively, they offer cooperation with companies for printing. These costs are also not included in their offered packages. The export tool of the three software applications, also the compatibility of 3D printers and different materials are not evaluated in this study and may be an issue for a next work.

A very important factor for successful and satisfying model surgery is the quality of DICOM data. Regarding radiation dose and metal artifacts due to brackets, CBCT has advantages compared with CT. But the quality of the rendered surfaces and segmented images is still better in thin-slice CT examinations. Therefore, the authors agree with other works that CT examinations with thin slices of the whole head are recommended to avoid defects in the rendered surface files [5, 13]. Although the supine patient position in the CT scanner may lead to altered soft tissue conditions and inaccuracy of soft tissue prediction. This may result in changes of soft tissue conditions and inaccuracy of soft tissue prediction. Also important is a good quality of 3D data of teeth scans or gypsum casts for merging with CT data [9]. This is similar in all three tested software applications.

### Strengths and limitations

As a strength, the authors mention that for evaluation of time consumption, all VSP was performed by one experienced person who had been trained on all three software applications. All work was done with the same PC.

A further strength is the chosen patient collective for this comparison. Nineteen already finished cases with available pre- and post-operative radiological 3D datasets were used for a realistic second planning of IQLFIIO, a complex surgical intervention. The surgical aim was the actually reached situation as found in the post-operative scans. We have successfully developed a standardized workflow for all three compared applications.

An aim of this study is to give an overview about the different costs and license products. These are different, but in similar ranges in all three software applications. It is difficult to perform and publish a price comparison because companies sometimes quickly change their price policies. This is a limitation of this study. However, the authors think that this work gives a suitable overview about the level of costs allowing to estimate usability in surgical planning routine.

For the system IPS Case Designer ®, the costs are outlined on the homepage of the company and apparent for everyone. For Dolphin Imaging ® and ProPlan CMF ®, the company has to be contacted to acquire cost information. One possible limitation of this study is that the authors only give a financial orientation instead of a price/performance ratio. However, it only seems to be possible to assess a price/performance ratio if the whole functional range is considered. This was not an objective of this work.

Generally, costs are higher; the more steps of the planning process have to be outsourced [6].

## Conclusion

Our study shows that all three tested software applications are usable for virtual IQLFIIO planning. Successful movement of bone segments and overlaying soft tissues proved feasibility. Time consumption and costs were found in similar ranges.

## Data Availability

The datasets used and/or analyzed during the current study are available from the corresponding author on reasonable request.

## Abbreviations

3D: Three-dimensional
CT: Computer tomography
CBCT: Cone beam computer tomography
DICOM: Digital Imaging and Communications in Medicine
IQLFIIO: Intraoral quadrangular Le Fort II osteotomy
PC: Personal computer
VSP: Virtual surgical planning
